# Land Degradation Caused by Construction Activity: Investigation, Cause and Control Measures

**DOI:** 10.3390/ijerph192316046

**Published:** 2022-11-30

**Authors:** Shubing Dai, Yulei Ma, Kuandi Zhang

**Affiliations:** 1 College of Water Resources and Architectural Engineering, Northwest A&F University, Yangling, Xianyang 712100, China; 2 Key Laboratory of Agricultural Soil and Water Engineering in Arid and Semiarid Areas, Ministry of Education, Northwest A&F University, Yangling, Xianyang 712100, China; 3 State Key Laboratory of Soil Erosion and Dryland Farming on the Loess Plateau, Institute of Soil and Water Conservation, Northwest A&F University, Xianyang 712100, China

**Keywords:** land degradation, soil erosion, construction activity, policy suggestions, control measures

## Abstract

The rapid expansion of construction land has been a common phenomenon worldwide, which resulted in the loss of high-quality arable land and severe land degradation. Here, a statistical analysis, together with a field investigation, was carried out in China to address the challenges. This study has gathered data on the reduction of land amount and quality caused by construction activities and has collected the relevant policies to control land deterioration caused by those activities. The increasing amount of farmland and open space are occupied by construction use. The annual growth of construction land from 2001 to 2017 was 43.64 × 10^4^ hm^2^, with an annual average of about 38 × 10^4^ hm^2^ of cultivated land being converted to construction land in China. Construction activities usually cause a deterioration of the physico-chemical properties in and around construction site soils. The organic matter of post-construction soil was lower than the pre-construction by 257.4~879.8%. A lack of strong economic incentives for developers, limited effectiveness of measures to control land degradation, and weak requirements and enforcement of relevant laws and regulations allow land degradation from construction activities to remain at a significant level. For more efficiency and success, the study proposes effective measures to control the hazards that occur so widely in China.

## 1. Introduction

Land can undertake a variety of functions, such as offering economic benefits, maintaining social stability, ensuring food security, and protecting the ecological environment [1,2]. With the rapid development of the economy, however, soil disturbance by construction activities, including occupation and erosion of large areas, has posed a threat to ecological and food security. Soil erosion rates in areas disturbed by construction activity are 2 to 40,000 times greater than pre-construction conditions, and soil erosion is an important component of nonpoint source pollution that degrades surface water quality [3,4]. Although land degradation caused by construction activities has significant on- and off-site environmental, economic, and social impacts, the lack of interest by academic geomorphologists in construction site erosion control might hamper vital research in this area [5].

Rapid economic growth in China over the last 30 years has resulted in massive construction activities. These constructions have altered landforms, vegetation, and waterways, thus leading to surface runoff, soil erosion, sedimentation, and land degradation [4]. From 1991 to 2003, the annual rate of land degradation caused by non-agricultural construction occupation in China exceeded 3800 km^2^, total area of land degradation of approximately 50 thousand km^2^, representing approximately 25% of the total amount of land resource degradation [6]. The area impacted by soil erosion from construction activity increased by 61.6 thousand km^2^, the amount of waste slag was 100.3 billion tons, and the amount of increased soil erosion was 10.1 billion tons between 2006 and 2010 in China [7].

Topsoil includes a plow layer (thickness of approximately 20 cm) and a plow pan (thickness of 6–8 cm). Topsoil is rich in organic matter, soil enzymes, and microorganisms and provides good nutrition and an environment to support plant growth. Processes of topsoil formation proceed very slowly, 1 cm of topsoil is formed under natural conditions every 300–400 years [8], 2.5 cm of topsoil is formed under cropland conditions every 200–1000 years, and even longer under pasture and forest conditions [9]. However, it is eroded in only one year, especially on construction sites. Construction activity degrades soil by loss, mixing, and compaction of the topsoil and subsoil [10]. For example, according to some estimates, from 1999 to 2004 large-scale infrastructure projects in Fujian, China, caused the loss of 1.76 million tons of topsoil during the construction phase and 0.43 million tons of topsoil during the first year of operation for each project [4]. Topsoil is generally removed during construction, and the surface soil following construction is often compacted and low in nutrients, providing poor growing conditions for vegetation [11]. Topsoil stripping, a current method used in international practice to protect topsoil resources, involves removing topsoil from the construction site just before construction, stockpiling it in a fixed location, and reapplying it to the site when construction is complete. Topsoil application promotes the establishment of a persistent vegetative cover and improves revegetation success [12,13]. Various countries have enacted laws and regulations to protect topsoil, such as the Topsoil Protection Act of Canada, the Good Practice Guide for Handing Soils of the United Kingdom, and the Surface Mining Control and Reclamation Act of the United States [13]. Meanwhile, China has also formulated corresponding laws and regulations that have stipulated topsoil stripping for construction projects, such as the soil and water conservation law of the People’s Republic of China and the Soil and Water Conservation Technical Specification of Development and Construction Projects. Increased regulatory requirements are increasing the number of construction sites where erosion control efforts are being implemented. However, there are still shortcomings in the act, policy, technical standards, and availability of funds for topsoil protection in China, resulting in topsoil loss from the construction site that remains serious [7].

The main aim of this study was to identify the main cause behind land degradation caused by construction activity through field investigations and statistical analyses. From there, effective measures to control the hazards that occur so widely in China were proposed.

## 2. Method and Materials

There are many different kinds of construction projects, and this study focuses on those that have a strong connection to soil and water conservation—construction projects will result in soil and water loss during the exploration and manufacturing phases. As a result, road, railway, and airfield construction projects are selected in the transportation sector, and well and opencast mining are chosen in the mineral development activities. Choosing the projects for crude oil and natural gas extraction, shipping, and storage in the fields that develop oil and gas resources, as well as water conservancy projects in infrastructure construction. Additionally, electricity transmission lines, and thermal, nuclear, and hydropower plants were selected for the electric power construction. Each type of project has a varied construction scale; hence, the amount of soil erosion produced varies substantially. Consequently, for the five types of construction projects described above, this study chose more than 500 projects from more than 1000 projects, and the chosen projects are representative of projects of this type as a whole (Table 1).

**Figure 1:**
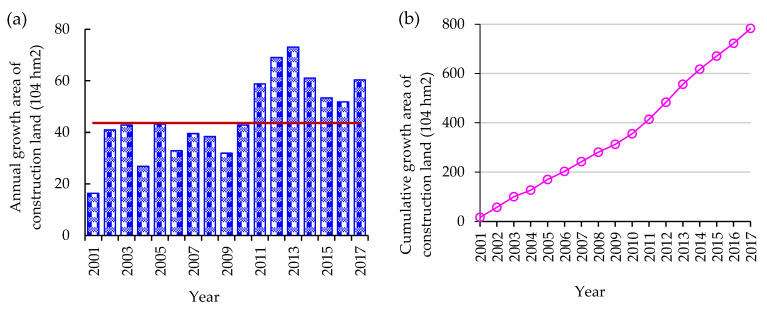
Annual growth area and cumulative growth area of construction land during 2001–2017. (**a**) Annual growth area, and (**b**) cumulative growth area.

The data on the land area for constructive activity, the number of construction projects, the area impacted by soil erosion from construction activity, and the amount of soil erosion caused by construction activity were obtained from bulletins, academic monographs, and journal articles. The annual land area occupied by constructive activity was obtained using data from the Bulletin of Land and Resources of China (Figure 1) [14]. The data for Figure 4 were gathered for the Bulletin of Soil and Water Conservation of China through field investigations and monitoring. It depicts the total number of construction projects and the region of responsibility for soil erosion control influenced by constructive activity [15]. Table 1 displays data from field investigations of more than 500 representative construction projects, including the total land area and the cultivated area occupied by construction activities, the area of land restoration, the volume of excavated and backfilled, and the volume of waste slag during construction [16]. The physico-chemical characteristics of site soil pre- and post-construction were gathered from field investigations, monitoring, and surveys of the representative constructive projects [17,18,19,20]. The Bouyoucos hydrometer method was used to determine soil texture. The organic matter content of the soil was calculated by multiplying the amount of soil organic carbon by 1.724, which was determined using the Walkley–Black method [20]. A pH meter was used to measure the pH of a soil/water (1:2.5) solution [19]. Soil pH, soil bulk density, organic matter, total nitrogen (N), and total phosphorus (P) content were determined using the potentiometric method, ring-cutting method, potassium dichromate oxidation–outer heating method, Kjeldahl method, and molybdenum–antimony anti-spectrophotometric method [17,18].

**Figure 2:**
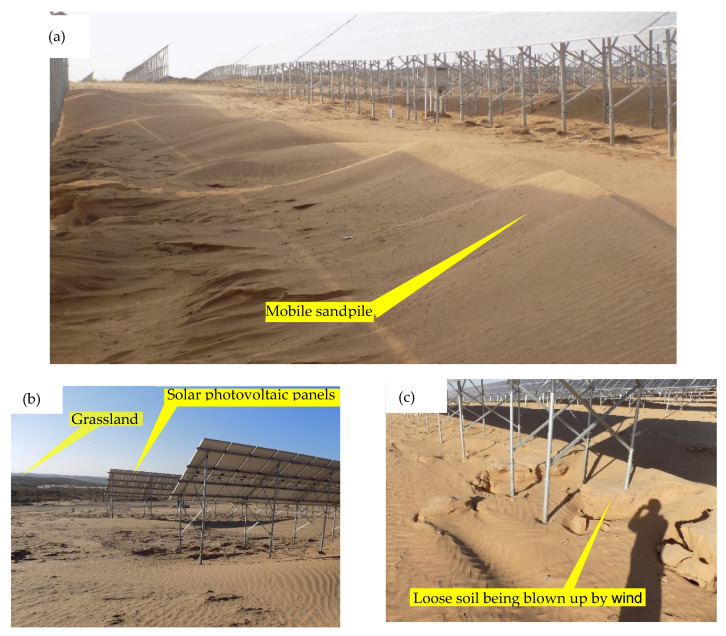
An onsite view of 25 October 2016, land desertification caused by the photovoltaic power generation project in Zhongning County, Ningxia Province. (**a**) Mobile dunes had been formed in the constructive area. (**b**) The land in the construction area was undergoing desertification, and the land outside the construction area was still grassland. (**c**) The soil around the support of the photovoltaic panel was blown away by the wind.

**Figure 3:**
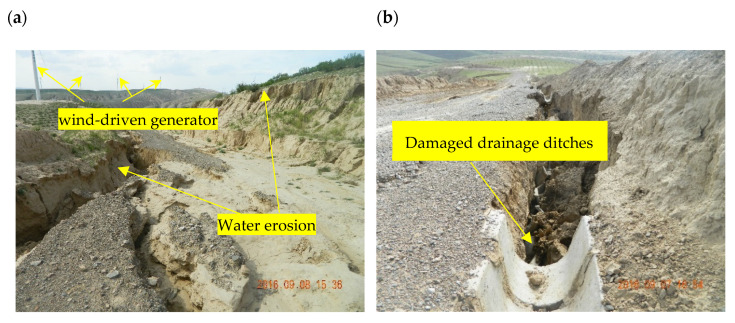

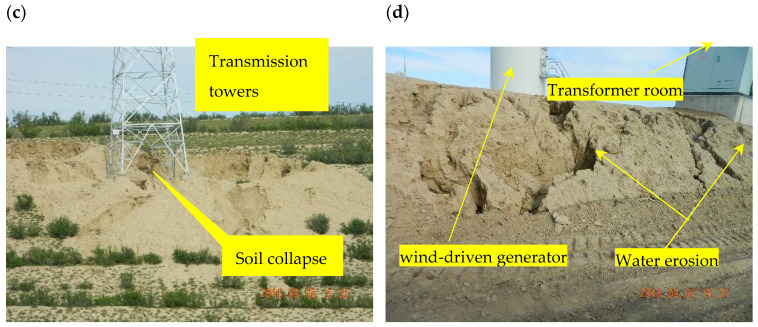
On-site view of 8 September 2016, water erosion caused by wind power project in Haiyuan County, Ningxia Province. (**a**) Water erosion destroyed the provisional road. The road was built to ensure that vehicles, personnel, and materials could be timely transported to the construction site during the construction of the wind power project. (**b**) Water erosion destroyed the drainage ditches. Because no protection measures had been implemented for the slope which was cut very steeply, resulting in serious water erosion and destroying the drainage ditches beneath the slope. (**c**) Soil collapse threatened the safety of transmission towers. (**d**) Water erosion occurred around the wind-driven generator and transformer room. If water erosion was not controlled, the safety of the wind-driven generator would be threatened.

To get a close look at the land degradation and soil erosion caused by constructive activities and to investigate the causes of such a serious issue on the construction site, the authors trekked to the site of the photovoltaic and wind power projects of Haiyuan and Zhongning County of Ningxia Province (Figure 2 and Figure 3).

## 3. Result

### 3.1. Decrease in the Number of Land Area

The pattern of reduction in the amount of land caused by construction activity included topsoil loss, excavation, and occupation, and the occupation was the most significant. As shown in Figure 1, the area of land used for construction increased by 741.70 × 10^4^ hm^2^ from 2001 to 2017, and the annual growth of construction land was 43.64 × 10^4^ hm^2^. Meanwhile, a large amount of agricultural land is converted to non-agricultural land every year. For example, from 2010 to 2013, an average of 38 × 10^4^ hm^2^ of agricultural land was occupied by construction land every year [14].

The total area of construction occupation, the area of land restoration following project completion, the area of permanent occupation, and the volume of material excavated, backfilled, and waste slag during construction were obtained from about 500 representative construction activities (Table 1). For line-type engineering, the land areas occupied by road, railway, oil and gas pipelines, and transmission lines engineering were 7.33, 5.09, 2.43, and 0.31 hm^2^ km^−1^. The areas of permanent occupation of the road, railway oil and gas pipelines, and transmission lines engineering accounted for 73, 68, 19, and 29% of the total construction areas, namely, the corresponding potential restoration areas amounted to 27, 32, 81, and 71% of the total construction area, respectively. Because the occupied lands have been permanently reserved as part of the construction project, the land use in these regions has changed as a result of soil structure being fully destroyed and land productivity is lost. Meanwhile, the proportion of cultivated land occupied by these linear activities ranged from 35% to 49%, and the cultivated land occupied by road construction was the most serious, at 3.60 hm^2^ km^−1^ (Table 1).

As shown in Table 1, for the block-type engineering, the areas occupied by a single project for opencast mining, hydraulic engineering, oil and gas chemical, hydropower plant, nuclear power plant, airfield construction, thermal power plant, and well mining were 1059.83, 986.75, 410.06, 350.57, 325.33, 261.50, 130.75, and 78.47 hm^2^, with the corresponding areas of permanent occupation accounted for 99%, 80%, 46%, 55%, 55%, 93%, 69%, and 87% of the total areas of construction activities, respectively. This means that the potential restoration areas amounted to 10% to 45% of the total construction areas, an average value of 27%. The area ratio of occupied cultivated land to the total construction area in the aforementioned block-type engineering ranged from 2% to 59%, with airfield construction engineering being the most significant, at 153.75 hm^2^.

*R*_3_ is expressed as the ratio in percentage between the area of land restoration and the total occupied area of construction engineering (Table 1)., With the exception of extraction and storage of oil and gas engineering, where the *R*_3_ is above 50%, all other engineering for block-type have an *R*_3_ below 50%, with opencast mining having the lowest *R*_3_ of 2%. The extraction and storage sites of oil and gas engineering are localized, the construction period is short, and the temporarily occupied land (57%) may be restored to its original state soon. However, *R*_3_ is just a purely theoretical value, and the temporarily occupied area cannot be timely reclaimed in real situations. [21] investigated that by the end of 2018, 40% of the total area of temporary occupied land had not been reclaimed in Hebei province from 2010 to 2017.

### 3.2. Decrease in the Land Quality

Construction activities, such as opencast mining and road construction, often led to a deterioration of the physico-chemical properties in and around the construction site soil. Through loss and compaction of topsoil, mixing of topsoil and subsoil, and occupation of the land by residual materials and waste materials, construction activities have potential long-term effects on the physico-chemical properties of the construction site soil, ultimately influencing land use and productivity. Table 2 portrays that the physical and chemical parameters of soil differed significantly in pre- and post-construction. [20] investigated that the very fine sand content of the soil in post-construction (6.91%) was significantly higher than that in pre-construction (0.82%), while the soil organic matter of pre-construction road was 3.57 folds higher than post-construction. Meanwhile, the soil bulk density in post-construction was greater than the pre-construction by 10.3%. Due to soil compaction during road construction, the soil bulk density increased [22]. Opencast mining practices have the potential to drastically alter landscape patterns and often result in a deterioration of soil’s physical and chemical properties [23]. [17] found that gangue spontaneous combustion had a significant effect on soil quality, as evidenced by the difference between the soil microbial biomass and organic matter content of 1552.77 × 10^5^ g^−1^ and 16.69 g kg^−1^ in the original landscape and their values after combustion of 10.35 × 10^5^ g^−1^ and 4.08 g kg^−1^. [18] also indicated that the organic matter content, total nitrogen (N), and total phosphorus (P) of the soil before mining were 9.79, 11.21, and 3.21 times higher than those after mining, respectively. If no land reclamation measures were implemented following opencast mining, the deterioration of the physical and chemical properties of the soil would be very serious. Due to the continuous production of opencast mining, the occupied land cannot be quickly restored to its original appearance and a significant portion of it is exposed for a long time, resulting in severe soil erosion. In addition, the waste mineral materials stored on the land negatively influenced the physical and chemical properties of the soil. Previous studies have shown that mine oils reclaimed to the forest, hay, and/or pasture developed soil organic carbon stocks comparable to those of undisturbed soils within 28 years of reclamation [24].

### 3.3. Soil Erosion Caused by Construction Activity

Soil erosion from construction sites is a major pattern of land degradation. Excavation and backfill disturbed the surface vegetation and generated waste soil during the construction period, resulting in serious soil erosion. As shown in Figure 2, the land leveling during the construction of the photovoltaic power generation project destroyed surface vegetation, resulting in the soil losing fine particles and gradually becoming sandy. As shown in Figure 3, the artificial landfill area was prone to water and gravity erosion during rainfall because of the bare surface and loose soil structure. In addition, the volume of newly added soil erosion was related to the volume of soil and rock displacement and abandoned soil and rock caused by construction engineering. According to some estimates, the amount of soil erosion produced by abandoned soil and rock can amount to 20~30% of the total amount of abandoned soil and rock [16]. As shown in Table 1, The volume of abandoned soils of a single-item project for opencast mining, hydraulic engineering, oil and gas chemicals, hydropower engineering, nuclear power plant airfield construction, thermal power plant, and well mining were 10,460.50, 201.60, 39.25, 818.48, 94.00, 11.67, 52.45, and 26.3 × 10^3^ m^3^. This implies that opencast mining, hydraulic engineering, and hydropower projects were the three major block-type engineering producing abandoned soil and rock, and opencast mining was the most significant. For each kilometer of length, the volumes of abandoned soil and rock associated with roads, railways, oil and gas pipelines, and electricity transmission lines were 3.36, 3.58, 0.06, and 0.02 × 10^4^ m^3^/km, respectively. That is to say, the volume of abandoned soil and rock produced by road and railway constructions was about 86.8 times that of oil and gas pipelines and transmission lines constructions. This implies that opencast mining and road construction generated the highest amount of soil erosion for block- and linear-type projects, respectively.

### 3.4. Policy Preventing Land Degradation Caused by Construction Activity

During construction work, the topsoil removed is one of the most valuable resources for land reclamation and ecological restoration in disturbed areas, as its contents in high concentrations of micro-organisms, nutrients, and native seeds [25,26]. Stripping topsoil also was applied to create high-quality land, thus alleviating the man-land contradiction. To control land degradation and soil erosion caused by construction activity, several laws, and norms enacted by China have made specific provisions for topsoil protection and utilization in construction activities, as shown in Table 3. The Land Administration Law of the People’s Republic of China issued in 1991 proposed that the cultivated land occupied by construction activities should be stripped of topsoil and used for re-cultivation. It was stipulated at the legal level regarding the system of farmland topsoil stripping and reuse as early as the revision of the Land Administration Law in 1998. However, the principled provisions were difficult to cope with the specific requirements in the promotion nationally, leading to a common dilemma that both local governments and construction businesses implementation dilemma from the specific situation to universal use. In 2004, the Land Management Law was revised to establish a balance of cultivated land occupation and compensation and to specify the responsibility of construction companies to protect the occupied land resources. The rapid expansion of construction land has caused a shortage of arable land resources. Hence, in 2006, China promulgated the Cultivate Land Requisition-Compensation Balance Policy. This policy stipulates that constructive enterprises must remove and store the cultivated layer soils from planned occupied and destroyed arable land to be used as materials for land development projects or arable land improvement. Meanwhile, the Soil and Water Conservation Law of the People’s Republic of China revised in 2010 also stipulates that the topsoil of arable land should be removed before construction. Topsoil stripping obligations are clearly defined in the Chinese Land Reclamation Regulations in 2011, and penalties for land reclamation obligors who do not perform topsoil stripping are also clearly defined.

China has made clear provisions for the protection and utilization of topsoil in construction projects at the level of laws and regulations and has also promulgated some technical specifications. In 2008, the Technical Standards for Soil and Water Conservation of Construction Projects stipulates that topsoil should be stripped and stored before the principal part of the project is constructed, and then used as cover soil for reclaimed cropland, woodland, and grassland after construction has been finished. In 2016, arable land was required to be fully stripped of the cultivated layer soils before being occupied, and standards for stripping the cultivated layer soils were proposed from a technical point of view.

## 4. Discussion

### 4.1. Urgency

China is developing rapidly. Construction activities are integral to this development, such as large-scale infrastructure development, highway and railway construction, and resource exploitation, and are advancing at unprecedented scale and speed. Such a dramatic change results in accelerated construction land growth becoming the dominant characteristic of land-use change in China [27]. As shown in Figure 1b, the construction land area increased by more than 7 million hectares during 2002–2012. Since 1978, the annual land use plan stands for the quota allocation of land converted to non-agricultural use has been carried out in China [28]. However, despite the construction land being strictly allocated and restricted by the Chinese government, the actual expansion rate reached 12.43% from 2006 to 2013, while expected 7.65% [28]. Ref. [29] investigated that the ratio of cultivated land converted to construction land has risen from approximately 60% during 1980–2010 to 67.5% during 2010–2015. Hence, how to control the amount of non-agricultural land converted from arable land is an urgent problem.

**Figure 4:**
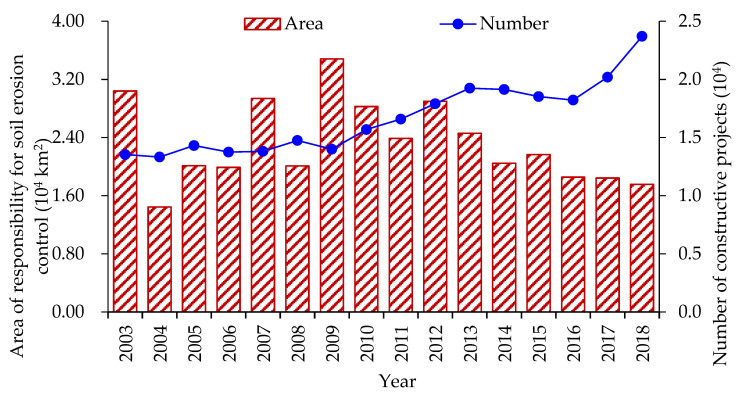
The area of responsibility for soil erosion prevention and control and the number of constructive projects in China from 2003 to 2018.

Our results also indicate that construction activities have the potential to increase soil erosion and cause a deterioration of soil’s physico-chemical properties (Table 1 and Table 2). As shown in Figure 4, the number of construction activities involving soil erosion was 42.67 × 10^4^ from 2003 to 2018, and the corresponding scope of soil erosion prevention and control responsibility was 23.22 × 10^4^ km^2^ [15]. This indicated that the number of construction activities remained at a high level and even an increasing trend, which caused a potential to increase soil erosion. Construction activities, such as opencast mining, have a long-term negative influence on the value of PH, organic matter content, and total N and P, in turn influencing land use and productivity (Table 2). Hence, there is an urgent need to control the degradation of the soil’s physico-chemical properties resulting from construction projects.

### 4.2. Cause behind Land Degradation

Land degradation has become a major issue in China due to rapid economic development and land-use change. There is a lack of strong economic incentive for the developer to control land degradation on construction sites. For a construction activity, the cost of preventing land degradation is relatively low, such as site erosion control, accounting for only 0.4% to 4% of the total project investment [16]. Generally, land degradation control on a construction site is perceived to be expensive and produces no clear return on investment in the construction industry. If land degradation control techniques are simply ignored by the developers, overall profits can be increased. Because the initial developer was only responsible for clearing and grading the site and putting roads and utilities, the final buildings and landscapes are frequently born by those who buy property on the site.

The effectiveness of the measures to control land degradation on site was limited. Although the measures of soil and water conservation have been designed and implemented to reduce erosion and trap sediment on-site in construction projects. However, the designers of these measures may not understand the erosion and sedimentation processes at the construction site. In addition, incorrect installation and maintenance, as well as the inability to apply them on time, have limited their effectiveness [5]. As shown in Figure 3a,b, despite the paving of the provisional road with gravel and supporting drainage measures, severe water erosion destroyed the provisional road and drainage ditches because the designer may not understand the local soil erosion environment. As shown in Figure 2, the construction area was undergoing desertification due to the complete clearing of the site. Low precipitation and a high potential for wind erosion make soils difficult to revegetate and reclaim after the construction of a photovoltaic power project. This indicates that problems can develop if the remediation is performed poorly or incorrectly.

The laws and regulations for preventing land degradation have been relatively weak in terms of requirements and enforcement. Although the Soil and Water Conservation Law requires the developer to remove the topsoil, there are no executable details as to how topsoil is removed and utilized and who provides the funds, which lacks practicability. In addition, the lax regulation of land use by local governments poses another major obstacle. Many construction activities in China ignore environmental laws because local governments tend to prioritize making money over protecting the environment [30].

### 4.3. Effective Measures and Policy Suggestions to Prevent Land Degradation

Due to the severe land degradation caused by construction projects, effective measures advocated by the national and local governments are urgently desired to control land degradation. Controlling land degradation caused by construction activity, such as land disturbance and site erosion, necessitates the motivations of the developer, requirements of the policy, knowledge of geomorphic processes, conditions, and experience in construction site management. The following effective measures and policy suggestions cover the main elements of construction site land degradation control.

Develop innovative and practical measures to improve land degradation control through a combination of academic geomorphologists and professional engineers using their integrative understanding of land degradation processes and construction site realities. Land degradation control plans and construction site management are often dominated by professional engineers with little involvement from academic geomorphologists. Regular site inspections and training of construction personnel can help avoid many potential erosion and slope stability problems, as well as ensure that land degradation control measures are properly installed and maintained. This is especially true if a geomorphologist specializing in land degradation control can be involved in the initial development design team.

Retain existing vegetation in parts of the site that remain undisturbed provides continuous and more effective protection from erosion than clearing the land and establishing new cover. This can be achieved by following the following three guiding principles. First, fit the construction to site conditions. If the ecology and terrain conditions of the construction site were not suitable for full clearing, then as much as possible the old vegetation was retained; otherwise, serious land degradation would be caused (Figure 2). Second, minimize surface disturbance during construction on native rangelands and forests. Third, remove topsoil on the plow and other available land types. Topsoil is one of the most valuable resources for ecological and arable land restoration, as it has high concentrations of micro-organisms, nutrients, and seeds.

Reduce bare soil exposure during and after construction. Soil erosion caused by wind and water is very susceptible to surface cover conditions. Generally speaking, most sites have pre-construction cover, such as agricultural, natural, or abandoned, which is usually removed or disturbed during construction, resulting in bare soil and causing soil erosion. Erosion can be dramatically decreased with minimized time between removing pre-construction cover and establishing post-construction cover. The second aspect of managing the construction schedule, especially in regions where there are obvious seasonal variations, is to construct whenever possible during the season of the year when the erosion potential is relatively low. Last but not least, using temporary cover to shield disturbed areas during construction can also reduce soil erosion.

Raise awareness of erosion control on construction sites and legal responsibility for land restoration among developers. The lack of an individual economic incentive for the construction business to control land degradation has limited the voluntary adoption of soil and water conservation measures. Therefore, strengthened regulatory requirements, combined with efforts to identify and publicize the benefits of erosion control, can help to alleviate land degradation by construction site erosion.

Conclusively, an effective measure to control land degradation on construction sites should be flexible, based on local characteristics, and should take into consideration temporal, spatial, and administrative factors as well as laws.

## 5. Conclusions

Land degradation has become a major issue in China due to rapid economic development and land-use change. Our study indicated a significant reduction of the land area due to construction activities and a large amount of agricultural land converted to non-agricultural land every year. In China, an average of 43.64 × 10^4^ hm^2^ of land was occupied by construction projects each year between 2001 and 2017, and an annual average of 38 × 10^4^ hm^2^ of agricultural land was converted to construction land from 2010 to 2013. Construction activities also have a significant influence on the soil’s physico-chemical properties, which in turn impacts land use and productivity. Construction site erosion is the main pattern of land quality degradation.

Serious land degradation caused by construction activities may be primarily attributed to the lack of strong economic incentives for developers, the limited effectiveness of measures, and the relatively weak requirements and enforcement of laws and regulations to control land degradation. Construction site erosion control is an important component of reducing and controlling land degradation and can be completed by developing innovative and practical measures for collaboration between design engineers, field engineers, and geomorphologists, reducing bare soil during and after construction, retaining existing vegetation where possible, and increasing regulatory requirements.

## Figures and Tables

**Table 1:** Comparison of the surface impacts of line-type engineering (per km) and block-type engineering (per single item).

Type of Constructive and Productive Engineering		Total Area of Construction Land	Cultivated Area of Construction Occupation	Permanently Occupied Area	Area of Land Restoration	*R*_1_	*R*_2_	*R*_3_	Volume of Soil and Rock Displacement	Volume of Abandoned Soil and Rock
		(hm^2^/km) or (hm^2^)	(hm^2^/km) or (hm^2^)	(hm^2^/km) or (hm^2^)	(hm^2^/km) or (hm^2^)				(10^4^ m^3^/km) or (10^4^ m^3^)	(10^4^ m^3^/km) or (10^4^ m^3^)
Line-type engineering	Road	7.33	3.60	5.38	1.95	0.73	0.49	0.27	17.60	3.36
	Railway	5.09	1.90	3.46	1.63	0.68	0.37	0.32	11.09	3.58
	Oil and gas pipeline	2.43	1.14	0.46	1.97	0.19	0.47	0.81	1.74	0.06
	Electricity transmission line	0.31	0.11	0.09	0.22	0.29	0.35	0.71	0.30	0.02
Block-type engineering	Opencast mining	1059.83	23.33	1048.08	11.75	0.99	0.02	0.01	18,127.25	10,460.50
	Hydraulic engineering	986.75	347.20	790.19	209.67	0.80	0.35	0.21	584.38	201.60
	Extraction and storage of oil and gas	410.06	82.25	188.13	221.94	0.46	0.20	0.54	548.19	39.25
	Hydropower plant	350.57	85.65	192.00	158.57	0.55	0.24	0.45	1689.35	818.48
	Nuclear power plant	325.33	124.33	180.33	145.00	0.55	0.38	0.45	1995.33	94.00
	Airfield construction	261.50	153.75	243.75	17.45	0.93	0.59	0.07	842.75	11.67
	Thermal power plant	130.75	45.65	90.28	40.46	0.69	0.35	0.31	269.56	52.45
	Well mining	78.47	22.68	68.18	10.29	0.87	0.29	0.13	126.74	26.39

Notes: The area of construction land, cultivated land and land restoration, and volume of soil displacement and abandoned soil and rock of line- and block-type projects were the average values per kilometer and single-item, respectively. *R*_1_ is the percentage ratio between the permanently occupied area and the total occupied area of construction engineering; *R*_2_ is the percentage ratio of the area of the occupied cultivated area to the total occupied area of construction engineering; *R*_3_ is the percentage ratio between the area of land restoration after construction completion and the total occupied area of construction engineering.

**Table 2:** Physico-chemical characteristics of site soil before and after construction.

References	Construction Activity	Soil Physico-Chemical Indicators	Physico-Chemical Indexes of Soil	
			Post-Construction	Pre-Construction
Safar, Kavian, & Parsakhoo, 2013 [20]	Road construction	Very fine sand content (%)	6.91	0.82
		Organic matter (%)	1.88	6.72
		Bulk density (g cm^−3^)	1.72	1.56
		Soil moisture (%)	35.24	48.16
Maiti, 2014 [19]	Opencast mining	PH	4.42	6.44
		Organic carbon (%)	1.53	1.16
		<2 mm soil fraction (%)	50.21	56.23
Ge et al., 2016 [18]	Opencast mining	PH	8.78	6.98
		Organic matter content (g kg^−1^)	3.56	34.88
		Total N (g kg^−1^)	0.14	1.57
		Total P (g kg^−1^)	0.42	1.33
Fan et al., 2010 [17]	Opencast mining	PH	7.58	8.42
		Bulk density (g cm^−3^)	1.10	1.38
		Organic matter content (g kg^−1^)	4.08	16.69
		Soil microbial biomass (10^5^ g^−1^)	10.35	1552.77

**Table 3:** Specific provision on topsoil protection and utilization of laws and norms in production and construction projects.

Date	Laws and Norms	Content Related to the Protection of Topsoil
Promulgated and implemented on 29 June 1991, and revised on 25 December 2010	*Soil and water conservation law of the People’s Republic of China and*	In the production and construction, try to balance excavation and filling, reduce the range of surface disturbance, and strip, store and reuse the topsoil of the occupied land.
Revised on 1 August 2004	*Land Administration Law of the People’s Republic of China*	Land destruction caused by excavation, subsidence, occupation, etc., land users should be responsible for reclamation in accordance with the relevant provisions of China; land users who do not have the conditions for reclamation or reclamation do not meet the requirements shall pay land reclamation fees, which were used exclusively for the land reclamation. dedicated to land reclamation. The reclaimed land shall be used first for agriculture.
Promulgated on 16 June 2006, and implemented on 1 August 2006	*China’s cultivate land requisition-compensation balance policy*	The developer who plans to occupy and destroy the cultivated land should strip the soil of the cultivated layer for soil improvement materials for land development projects or other arable land soil improvement.
Implemented on 1 July 2008	*Soil and Water Conservation Technical Specification of Development and Construction Projects*	Before starting the principal part of the project, the topsoil should be stripped and then stacked together and used as the cover soil for the reclaimed cultivated land, forest, and grassland after the construction is finished.
Promulgated and implemented on 8 January 2011	*China’s regulations on the protection of prime farmland*	The constructive companies that plan to occupy and destroy the prime farmland should strip and reuse the cultivated layer of the prime farmland.
Promulgated and implemented on 5 March 2011	*China’s regulations on the Land reclamation*	The topsoil stripping of the arable, forest, and pastureland planned to be occupied and destroyed is needed, and then the stripped topsoil is used for the reclamation of the damaged land. It is worth noting that topsoil polluted by heavy metal pollutants or other toxic and harmful substances shall not be used as reclaimed materials.
Promulgated and implemented on 8 June 2016	*Technical Standards for Soil Stripping and Reusing the Cultivated-layer soil*	The stripping scope of the plow layer was defined and the technical process and standards of stripping, transportation, storage, and reply of plow layer soil were regulated.

## Data Availability

All data included in this study are available upon request by contact with the corresponding author.
